# GenAI and Faculty Collaboration Support Feasible Development of Curriculum-Aligned Open-Access Board-Style Questions

**DOI:** 10.1007/s40670-025-02472-y

**Published:** 2025-07-31

**Authors:** Ronaé McLin, Colleen M. Croniger, Amy L. Wilson-Delfosse

**Affiliations:** 1 Case Western Reserve University School of Medicine, Cleveland, OH 44106 USA; 2 Department of Nutrition, Case Western Reserve University School of Medicine, Cleveland, OH 44106 USA; 3 Office of Medical Education, Northeast Ohio Medical University College of Medicine, Rootstown, OH 44272 USA

**Keywords:** Anatomy education, Generative AI, Assessment

## Abstract

A curriculum-aligned generative AI chatbot was developed to create USMLE-style anatomy multiple-choice questions using open-access resources. Subject matter experts evaluated 100 of these questions and 83% were found to be potentially usable, with many needing minimal or no modifications. Sentiment analysis of subject matter expert interviews showed mild positive sentiment while acknowledging both advantages and limitations of AI-generated assessment items. Interestingly, the chatbot revealed discrepancies between faculty expectations and documented learning objectives, highlighting opportunities for curricular improvement. This ethically designed system provides a scalable, cost-effective approach to enhance equity and support local assessment development in medical education.

## Background

Medical education increasingly emphasizes preparation for licensing examinations such as the United States Medical Licensing Examination (USMLE) Steps 1 and 2 CK, driving students toward third-party question banks and resources. While these resources often provide comprehensive question sets, their high cost can create inequities among students, particularly disadvantaging those with fewer financial resources [1, 2]. Their generalized nature often leads to poor alignment with institutional curricula, which can diminish the effectiveness of local educational efforts and cause students to overlook key curricular objectives [3].

Recent advances in generative artificial intelligence (AI), especially large language models such as OpenAI’s ChatGPT, have emerged as potential solutions for creating customized, high-quality, curriculum-aligned assessment items [4–7]. ChatGPT, for instance, has demonstrated significant capabilities by achieving scores at or near passing levels on USMLE Steps 1, 2 CK, and 3 without explicit medical training, suggesting its substantial potential to assist in medical education assessment development [5].

Although promising, integrating AI into assessment design presents important challenges that must be addressed to ensure its effective and ethical use in medical education. Ethical concerns such as potential copyright violations, transparency of content sources, and quality assurance processes must be explicitly addressed [8]. Ensuring that AI-generated content is both accurate and pedagogically relevant necessitates rigorous faculty involvement and validation. While many medical educators lack formal training in writing USMLE-style multiple-choice questions (MCQs), they bring deep subject matter expertise. When combined with generative AI tools, these educators can effectively contribute to the creation of high-quality, curriculum-aligned assessments, leveraging AI capabilities without needing to master the nuances of item writing themselves [9].

To explore these possibilities, we developed and evaluated a generative AI–driven chatbot designed to create ethically sourced, curriculum-aligned MCQs in anatomy.

Our investigation aims to evaluate both the quality of AI-generated MCQs through expert review and the subjective experiences of faculty in editing these items. Understanding these dimensions is crucial for determining the practicality, efficacy, and acceptability of generative AI tools in medical education contexts. By offering a reproducible and ethically sound model, we seek to demonstrate a scalable solution that can enhance equity, reduce costs, and strengthen curricular alignment, particularly in resource-constrained educational situations.

## Activity

As it permitted the creation of a custom chatbot, a subscription version (ChatGPT Plus) of OpenAI’s ChatGPT-4o was selected to power a chatbot for generation of MCQs styled like USMLE board exam questions, specifically clinical vignette-based MCQs. The chatbot was instructed to consider the USMLE Content Outline, which was uploaded to the chatbot’s knowledge bank, and to limit generative content to the USMLE Physician Competency: identifying the underlying anatomic structure, cell/tissue structure, or physical location [10]. Each item drew from at least two open-access references, cited in answer explanations, to ensure ethical use of published resources. The chatbot also accessed Openverse and the National Library of Medicine’s Open-i API for inclusion of open-license images in selected questions. The chatbot was directed to utilize these open-access resources to avoid any copyright infringements.

To reinforce the local curriculum, the chatbot-generated MCQs directed users to specific sections of the anatomy textbook, *Moore’s Clinically Oriented Anatomy, 8th edition*, recommended by Case Western Reserve University School of Medicine (CWRU SOM) for further study [11]. Additionally, learning objectives from a 10-week segment of the longitudinal anatomy curriculum that runs parallel to an early first-year integrated systems course that includes endocrine and reproductive subject areas were uploaded along with a related faculty-developed study guide to assure alignment with local course content. The chatbot was prompted to create a test blueprint based on the included anatomy subject areas covered in the curriculum and then prompted to create 20 MCQs evenly distributed across the selected content areas.

Three subject matter experts (SMEs) in the areas of anatomy (PhD trained), radiology (clinically trained), and ultrasound (clinically trained) reviewed the AI-generated MCQs for correctness, quality, clinical relevance, and curricular alignment. Edits were tracked in a Microsoft Word document. The authors individually reviewed the edits and comments that the SMEs made about each question and agreed on themes that described types of edits made. The authors then individually categorized edit types for each question and met again to achieve consensus.

Author RM conducted structured interviews with SMEs and subjected the transcripts of these interviews to a sentiment analysis using R (version 4.4.2) “sentimentr” package [12]. This tool compares words to a dictionary of polarized terms, assigning + 1 for positive words and − 1 for negative words, and then adjusts these scores based on contextual modifiers such as negators, amplifiers, de-amplifiers, and adversative conjunctions. Sentiment scores below 0 indicate negative sentiment, above 0 indicate positive sentiment, and around 0 indicate neutrality.

## Results

One hundred MCQs were generated for the 10-week anatomy segment under consideration in the areas of the female breast, general genitourinary system, female genitourinary system, male genitourinary system, and superficial neck/thyroid gland. Questions were categorized as potentially usable or not usable.

### SME Editing Results

Eighty-three percent of the questions were determined to be factually correct and potentially usable. Among these, 40% (*n* = 33/83) required no revision, and 22% (*n* = 18/33) needed minor edits for clarity. The remainder were not used because SMEs disliked the question structure or found them misaligned with the curriculum, particularly for the female genitourinary and superficial neck/thyroid gland topics (Table 1).

**Table 1:** Perception of chatbot-generated MCQs by subject matter experts

	%	*N*
**Assessment status** (total = 100)		
Potentially usable questions	83%	83
Not usable questions	17%	17
**Potentially usable questions** (total = 83)		
No revision needed	40%	33
Minor revision	22%	18
SME perceived the question to be misaligned	23%	19
SME did not like the structure of the question	16%	13
**Potentially usable by topic** (total = 83)		
Female breast	95%	19
Female genitourinary	65%	13
Male genitourinary	100%	20
General genitourinary	85%	17
Superficial neck/thyroid gland	70%	14

Overall, 17% of the MCQs were deemed not usable, primarily due to factual inaccuracies. The rest were discarded because they included answers in the question stem or were too ambiguous for a correct response (Table 1).

### Sentiment Results

#### Overall Impression

Two of the three SMEs were interviewed and reported an average sentiment score of 0.26 (SD = 0.35), reflecting a mildly positive view of the AI-generated MCQs (Table 2). They considered the questions moderately good in quality and appreciated the clinical vignettes for relevance and realism, although there were concerns that the clinical complexity exceeded preclinical students’ readiness.

“[MCQs] were mostly clinically-based questions which is good because it contextualizes the information for the students, but I think they were just a little bit too advanced for first and second year medical students who do not have the clinical background yet.”

**Table 2:** Sentiment analysis

Category	Mean sentiment score	Standard deviation	Interpretation
Overall impression	0.26	0.35	Mildly positive
Facilitation of work	− 0.05	0.28	Neutral to slightly negative
Alignment to curriculum	− 0.07	0.16	Neutral to slightly negative
AI’s potential	0.17	0.34	Slightly positive

#### Facilitation of Work

SMEs reported an average sentiment score of –0.05 (SD = 0.28), reflecting a neutral to slightly negative view of the chatbot’s usefulness in creating assessment questions and found the MCQs helpful because they provided a starting point for creating such resources. AI-generated content became especially helpful if an SME felt they did not possess a particular skill set to generate certain types of questions.

“I can tell you that I could never have done this without these AI generated questions because I cannot write clinical scenarios, and I don’t feel qualified.”

#### Alignment to Curriculum

When evaluating alignment with the anatomy curriculum, SMEs reported an average sentiment score of –0.07 (SD = 0.16), indicating a neutral to slightly negative impression. A recurring theme during editing and interviews was that SMEs felt the anatomical concepts in the AI-generated MCQs did not accurately reflect the curriculum.

“There were some significant gaps between questions and what is being taught in the curriculum and we cut those [MCQs]… So many times,the other SMEs would say, ‘no, I don’t teach it’.”

Because the chatbot was trained on the published learning objectives and study materials provided to students, by design, its output accurately reflected the guidance students received.

#### AI’s Potential

SMEs reported a slightly positive average sentiment score of 0.17 (SD = 0.34), recognizing the value of AI-generated MCQs in saving time and allowing educators to focus on areas needing more attention.

“For a teacher who is also a clinician, it may be challenging for them to find the time to do a lot of this on their own.”

To reduce resistance, it is important to involve educators early in the AI creation process. Their input helps build trust and ensures alignment with curricular goals.

“I think including educators in the process of coming up with the queries that we’re inputting into the language learning models would be potentially useful in getting more relevant questions to the course.”

## Discussion

By leveraging only open-access resources, our chatbot model offers a tangible path toward more equitable assessment development in medical education. This approach avoids copyright issues and enables scalable, cost-effective solutions, especially valuable in under-resourced environments. As Hilton [13] and Wartman and Combs [14] note, open educational resources and AI-supported tools can help level the playing field for learners lacking access to commercial content.

A key takeaway from our process is that the value of generative AI in education increases with faculty involvement. Educators were essential in reviewing and refining questions, ensuring accuracy and alignment with institutional goals. This supports findings from implementation science, which highlights that stakeholder engagement is often essential to the sustained success of educational innovations [15].

SME feedback highlighted a secondary benefit of the chatbot: identifying misalignments between faculty expectations and documented curricular content. This kind of discrepancy, well-documented in the curriculum mapping literature, can inform targeted quality improvement by highlighting areas in need of clarification, alignment, or instructional revision [16].

This project had limitations. Reliance on open-access content, while ethical, can limit topic depth and breadth. The study’s scope was also narrow, involving only three subject matter experts from one institution and focusing solely on anatomy, limiting generalizability.

Generative AI holds considerable promise for medical education, but its success depends on thoughtful integration. These tools should augment, not replace, faculty expertise by generating draft content that educators can refine. As part of a broader shift toward sustainable, inclusive, and data-informed educational practices, AI-assisted assessment creation offers scalable potential.

Notably, Table 1 indicates a variation in the perceived usability of AI-generated questions across different anatomical topics. While this study did not definitively determine the reasons for this variability, several factors could contribute. For example, there may have been differences in the depth, quality, or clarity of the open-access resources and specific curricular materials that the AI chatbot utilized for each topic; there may have been inherent differences in the capabilities of the generative AI model with regard to accurately synthesizing and formulating questions for different anatomical regions; or there may have been a more pronounced divergence between documented learning objectives and subject matter expert expectations for certain topics, as was observed more generally in our findings. Further investigation could elucidate the specific interplay of these elements and help refine AI-assisted content generation for more challenging subject areas.

Additional research also will be needed to examine whether AI-generated assessments influence learning outcomes, summative performance, and long-term retention, as well as explore faculty and student trust in these tools. With continued development and feedback, generative AI can support the human expertise that remains at the core of medical education.

## Data Availability

The dataset supporting the conclusions of this study is not publicly archived because it is small and context‑specific.
